# Preparation of Biomorphic Porous SiC Ceramics from Bamboo by Combining Sol–Gel Impregnation and Carbothermal Reduction

**DOI:** 10.3390/polym11091442

**Published:** 2019-09-02

**Authors:** Ke-Chang Hung, Tung-Lin Wu, Jin-Wei Xu, Jyh-Horng Wu

**Affiliations:** 1 Department of Forestry, National Chung Hsing University, Taichung 402, Taiwan; 2 College of Technology and Master of Science in Computer Science, University of North America, Fairfax, VA 22033, USA

**Keywords:** bamboo, carbothermal reduction, ceramic, silicon carbide, sol–gel process

## Abstract

This study investigated the feasibility of using bamboo to prepare biomorphic porous silicon carbide (bio-SiC) ceramics through a combination of sol–gel impregnation and carbothermal reduction. The effects of sintering temperature, sintering duration, and sol–gel impregnation cycles on the crystalline phases and microstructure of bio-SiC were investigated. X-ray diffraction patterns revealed that when bamboo charcoal–SiO_2_ composites (BcSiCs) were sintered at 1700 °C for more than 2 h, the resulting bio-SiC ceramics exhibited significant β-SiC diffraction peaks. In addition, when the composites were sintered at 1700 °C for 2 h, scanning electron microscopy micrographs of the resulting bio-SiC ceramic prepared using a single impregnation cycle showed the presence of SiC crystalline particles and nanowires in the cell wall and cell lumen of the carbon template, respectively. However, bio-SiC prepared using three and five repeated cycles of sol–gel impregnation exhibited a foam-like microstructure compared with that prepared using a single impregnation cycle. Moreover, high-resolution transmission electron microscopy and selected area electron diffraction revealed that the atomic plane of the nanowire of bio-SiC prepared from BcSiCs had a planar distance of 0.25 nm and was perpendicular to the (111) growth direction. Similar results were observed for the bio-SiC ceramics prepared from bamboo–SiO_2_ composites (BSiCs). Accordingly, bio-SiC ceramics can be directly and successfully prepared from BSiCs, simplifying the manufacturing process of SiC ceramics.

## 1. Introduction

Over the past few decades, silicon carbide (SiC) ceramics have been extensively used in the structures of modern aviation vehicles, such as rocket nozzles, aeronautic jet engines, and aircraft brake materials, because such ceramics exhibit remarkable physical properties, including wear, corrosion, and thermal resistance [1,2,3,4,5]. In particular, SiC ceramics hold considerable promise for use as solar energy absorbers. Solar-energy-absorbing devices require ceramics with open porosity, excellent solar energy absorption performance, and high thermal conductivity. In general, SiC ceramics that exhibit larger grain sizes and are supplemented with α-SiC can produce relatively high thermal conductivity levels [6].

Reaction processing of SiC is a technique for producing advanced SiC-based structural ceramics. This technique involves a chemical reaction between carbon (C) and silicon (Si) during sintering [5]. In recent years, the use of wood as a carbon template for preparing SiC ceramics with a wood-like microstructure (wood-like SiC ceramics) has emerged as a relatively new research area [6,7,8]. Therefore, several techniques have been developed for fabricating porous wood-like SiC ceramics, including reactive infiltration with Si-containing melts [9,10], reactive silicon vapor infiltration [11,12,13], and SiO_2_ sol–gel impregnation combined with carbothermal reduction [14,15,16,17,18,19,20,21,22]. In the process of SiO_2_ sol–gel impregnation combined with carbothermal reduction, the charcoal is impregnated with SiO_2_ sol at ambient temperature under reduced pressure. Subsequently, the water initiates the hydrolysis reaction of SiO_2_ sol and then polycondensation reaction by dehydration or dealcoholation. The sols within charcoal are gelation as gels in 105 °C to prepare charcoal–SiO_2_ composites [23], and then the charcoal–SiO_2_ composites were subjected to carbothermal reduction reactions for fabricating SiC ceramics. Therefore, this technique has several advantages, for example, it is a low-cost approach, involves easy synthesis procedures, involves relatively low temperatures of synthesis, provides high-purity resultant products, and can retain the structure and morphology of starting carbonaceous materials [21].

Bamboo, a perennial woody plant belonging to the Gramineae family, is widely distributed across Asia [24,25,26] and exhibits higher growth rates than do other woody plants [27,28,29]. In Taiwan, bamboo is extensively used as a raw material for handicraft, furniture, and construction applications [30,31]. Bamboo materials are similar to wood, and they have a porous structure that renders them useful for realizing different penetration and treatment procedures [32]. Studies have focused on the crystalline-phase composition of wood-like SiC ceramics. The use of bamboo as an industrial raw material can improve economic efficiency. Accordingly, this study explored the feasibility of using bamboo to produce porous biomorphic SiC (bio-SiC) ceramics by combining sol–gel impregnation and carbothermal reduction. In addition, the study investigated the effects of sintering temperature, sintering duration, and repeated cycles of sol–gel impregnation on the crystalline phase and microstructure of the bio-SiC ceramics.

## 2. Materials and Methods

### 2.1. Experimental Materials and Procedure

**Figure 1:**
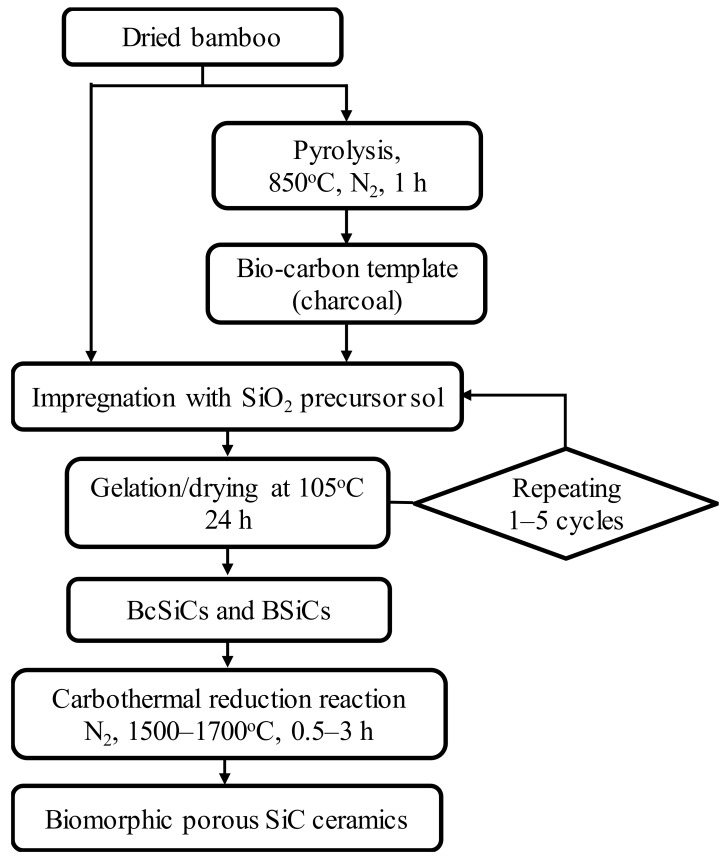
Processing scheme for manufacturing silicon carbide (SiC) ceramics with a biomorphic pore structure from bamboo by combining sol–gel impregnation and carbothermal reduction.

This study obtained 4-year-old moso bamboo (*Phyllostachys pubescens* Mazel) from a local bamboo-processing factory (Nantou, Taiwan). The obtained bamboo was air-dried and then cut into rectangular specimens measuring approximately 50 mm (L) × 25 mm (T) × 5 mm (R). Each of the bamboo specimens was converted to a porous biocarbon template (bamboo charcoal) through carbonization in a nitrogen atmosphere at a temperature of 850 °C, heating rate of 5 °C/min, and holding time of 1 h. A SiO_2_ precursor sol was formulated by mixing methyltrimethoxysilane (MTMOS) (Acros Chemical, Geel, Belgium) and methanol at a molar ratio of 0.13:1. In the sol–gel process, the bamboo and bamboo charcoal specimens were impregnated with the prepared sol under reduced pressure for 2 days. The impregnated specimens were then placed in an oven controlled to 105 °C for 24 h to age the gels. Repeated impregnation cycles (one, three, and five cycles) were applied, producing various bamboo charcoal–SiO_2_ composites (BcSiCs) and bamboo–SiO_2_ composites (BSiCs). Similar to the wood-SiO_2_ composites reported in our previous study [33], the Fourier transform infrared (FTIR) absorption bands at 1270 (Si–CH_3_, ν), 1104 (Si–O–Si, δ), and 778 cm^−1^ (Si–O–C, ν) were clearly observed in these BcSiCs and BSiCs (data not shown). This result confirms that silica mixtures are also formed in the bamboo-based composites. Consequently, the derived BcSiCs and BSiCs were subjected to carbothermal reduction reactions in nitrogen atmosphere (flow rate: 2.5 L/min) in a heater furnace (Nabertherm, LHT 04/17 SW, Lilienthal/Bremen, Germany) at 1500–1700 °C for 0.5–3 h to form porous bio-SiC ceramics. Figure 1 presents a summary of the processing scheme for the preparation of the porous bio-SiC ceramics.

### 2.2. Characterization

The crystalline phases of bamboo charcoal and porous SiC ceramics were characterized through a powder X-ray diffraction (XRD) instrument (MAC Science, MXP18, Tokyo, Japan) operated in the 2θ range of 2–35° using CuKαl (λ = 1.5406 Å) radiation at 40 kV and 30 mA. The morphology and detailed structural features of the specimens were determined using a scanning electron microscopy (SEM) instrument (JEOL, JSM-7401F, Tokyo, Japan) and a high-resolution transmission electron microscopy (HRTEM) instrument (JEOL, JEM-2100F, Tokyo, Japan), respectively. Field-emission measurements for the nanowires of the synthesized SiC were performed in a vacuum chamber at a pressure of approximately 5.0 × 10^−7^ Torr. A thermal analyzer (PerkinElmer, Pyris 1, Shelton, CT, USA) was used to elucidate the synthesis and oxidation mechanisms of the samples. Thermogravimetric analysis (TGA) was conducted in an air atmosphere from 50 to 950 °C at a heating rate of 10 °C/min.

## 3. Results and Discussion

### 3.1. Effect of Sintering Temperature on the Properties of Porous Biomorphic Silicon Carbide (Bio-SiC) Ceramics

**Figure 2:**
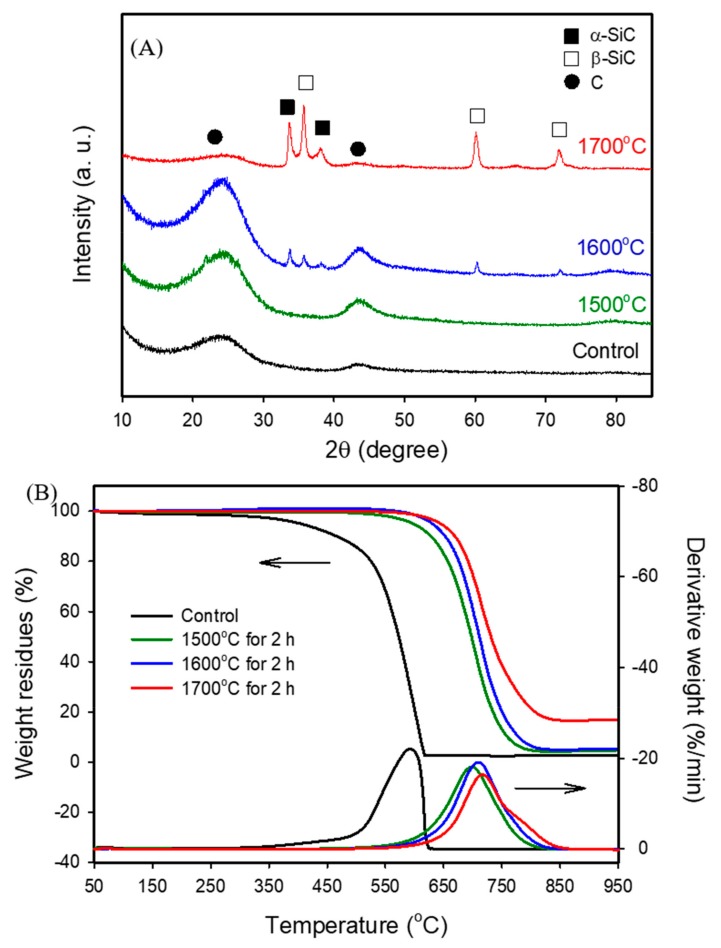
X-ray diffraction (XRD) patterns (**A**) and thermogravimetric analysis (TGA)/derivative thermogravimetry (DTG) curves (**B**) of BcSiCs sintered at different temperatures for 2 h.

To understand the effect of sintering temperature on the properties of the porous bio-SiC ceramics fabricated in this study, the following test conditions were applied: impregnation of bamboo charcoal with a SiO_2_ precursor sol (BcSiC) in a single cycle and then sintering at different temperatures for 2 h. Figure 2A illustrates the XRD patterns of bamboo charcoal and the BcSiCs sintered at 1500, 1600, and 1700 °C for 2 h. When the samples were sintered at temperatures below 1700 °C, two broad carbon diffraction peaks could be clearly observed at 2θ values of approximately 24° and 43°, which were attributed to the (002) and (101)/(100) planes of carbon, respectively. These results indicate that the samples had some unreacted carbon [3,21]. However, both specific carbon peaks almost completely disappeared after the samples were sintered at 1700 °C. Furthermore, when the samples were sintered at 1600 and 1700 °C, peaks associated with the α-SiC (2θ = 33.6° and 38.1°) and β-SiC phases (2θ = 35.3°, 60.0°, and 71.8°) were observed, and the intensity of the peaks increased with the sintering temperature. Similar observations were reported by Qian and Jin [21], who suggested that preparing β-SiC through carbothermal reduction usually results in a minor amount of α-SiC.

**Figure 3:**
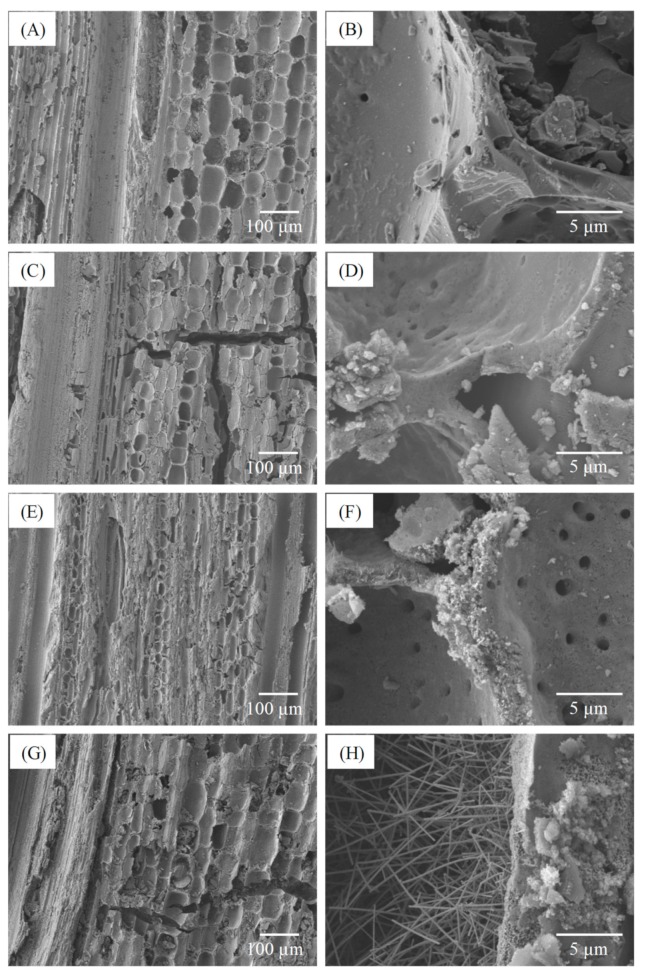
Scanning electron microscopy (SEM) micrographs of bamboo charcoal (**A**,**B**) and BcSiCs sintered at 1500 °C (**C**,**D**), 1600 °C (**E**,**F**), and 1700 °C (**G**,**H**) for 2 h.

Figure 2B presents the TGA curve of bamboo charcoal, indicating a considerable decrease in weight residue at sintering temperatures higher than 300 °C in an air atmosphere. A weight residue of only 2.7% was observed when the sintering temperature was up to 620 °C, this is because the carbon substrate was completely thermally degraded at approximately 600 °C [34]. By contrast, for the BcSiCs sintered at 1500, 1600, and 1700 °C, the thermal degradation onset temperatures were nearly 520, 600, and 610 °C, respectively. When the sintering temperature reached 950 °C, the weight residues of the three sintered BcSiCs were 4.7%, 5.3%, and 17.0%. Figure 2B also presents the derivative thermogravimetry (DTG) curves of the samples. As shown in the figure, the maximum thermal degradation temperature of bamboo charcoal was 590 °C, whereas the maximum thermal degradation temperatures of the BcSiCs sintered at 1500, 1600, and 1700 °C were 695, 700, and 710 °C respectively, signifying that the thermal stability of the BcSiCs could be improved by increasing the sintering temperature. The primary reason for this phenomenon is that SiC was generated through the carbothermal reduction of bamboo charcoal and SiO_2_ during high-temperature sintering. A higher sintering temperature typically results in a more complete reaction and thus, a more thermally stable SiC ceramic. Figure 3 depicts the SEM micrographs of the BcSiCs sintered at 1500–1700 °C for 2 h. According to these micrographs, all specimens retained the inherently porous microstructure of bamboo, and the resulting bio-SiC ceramics replicated the original texture of the carbon template (Figure 3C–H). After the sintering process, SiC grains were prominently formed on the cell wall and roughened the cell wall surface. However, among the specimens, only the BcSiC specimen sintered at 1700 °C formed a large amount of SiC nanowires on the cell wall (carbon template) surface (Figure 3H), and the obtained SiC nanostructures comprised a nanowire core with extensional platelets. A similar phenomenon has been observed by Ding et al. [34] and Hata et al. [35]. Accordingly, 1700 °C was considered the optimum sintering temperature for the preparation of bio-SiC ceramics in this study.

### 3.2. Effect of Sintering Duration on the Properties of Porous Bio-SiC Ceramics

**Figure 4:**
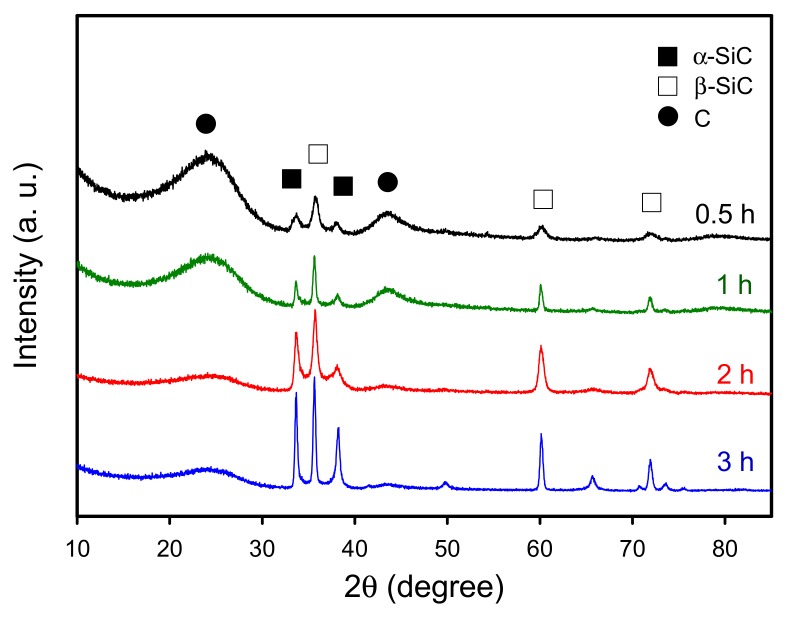
XRD patterns of BcSiCs sintered at 1700 °C for different durations.

To understand the effect of sintering duration on the properties of porous bio-SiC ceramics, this study applied the following test conditions: impregnation of bamboo charcoal with the SiO_2_-precursor sol (in one cycle) and then the execution of sintering at 1700 °C for different durations. Figure 4 shows the XRD patterns of the BcSiCs sintered at 1700 °C for 0.5–3 h. For the specimen prepared at 1700 °C for 0.5 h, five distinct diffraction peaks of α- and β-SiC were observed along with two major peaks of carbon. However, as the sintering duration increased, the intensity of the SiC-associated peaks increased, whereas that of the carbon-associated peak decreased. This result is similar to that reported by Locs et al. [6], who demonstrated that prolonged sintering positively influenced SiC formation. After 2 h of sintering, the peak of carbon nearly disappeared, whereas the peaks of the SiC crystal phase were clearly observed. A similar result has also been reported by Locs et al. [6] and Qian and Jin [21], when a sufficient reaction time was allocated, a favorable reaction between carbon and SiO_2_ was achieved. Accordingly, a well-crystallized bio-SiC ceramic could be produced through sintering at 1700 °C for more than 2 h. 

In addition, comparison with previously reported XRD data for wood-derived porous SiC ceramics [1,3,6,15,16,17,18,21,22], this study shows that more α-SiC (hexagonal type) is formed in bamboo-derived porous SiC ceramics. Locs et al. [6] reported that the presence of different impurities (e.g., Ca, K, Na, etc.) in the biocarbon matrix could promote the formation of high-temperature SiC crystal phases (α-SiC). Generally, bamboo has higher alkaline extractives, ash, and silica contents as compared to wood [36,37]. Accordingly, the sintered BcSiCs form more α-SiC than wood-derived porous SiC ceramics, which can be explained as the bamboo charcoal is rich in alkaline metals and mineral substances.

**Figure 5:**
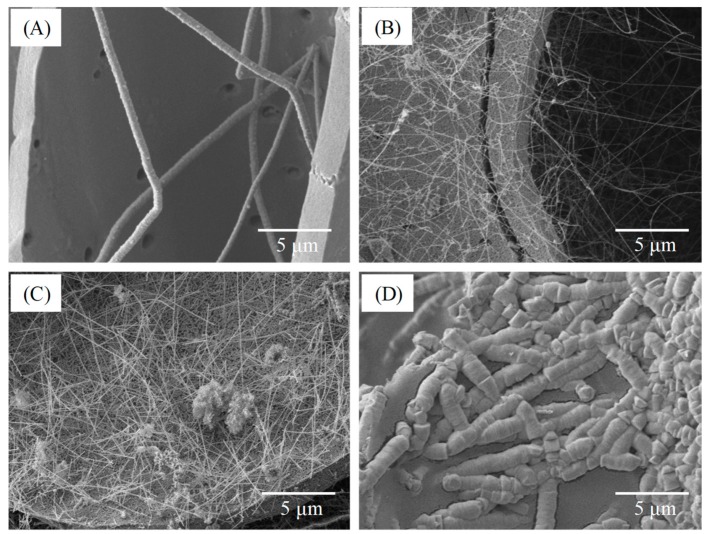
SEM micrographs of BcSiCs sintered at 1700 °C for 0.5 h (**A**), 1 h (**B**), 2 h (**C**), and 3 h (**D**).

Figure 5 depicts the SEM micrographs of the BcSiCs sintered at 1700 °C for different durations. Observing the microstructure of the specimen sintered for 0.5 h (Figure 5A) revealed that the cell wall surface was similar to the smooth surface of bamboo charcoal, but a small amount of SiC nanowires were formed in the cell lumen. However, when the sintering duration was extended to 1–2 h, the cell wall surface became rough and the cell lumen comprised a higher amount of SiC nanowires (Figure 5B,C). After 3 h of sintering, this study observed the formation of a large amount of SiC crystal particles and nanowires on the surface of the specimen (Figure 5D). Additionally, the SiC nanowires in this specimen were significantly shorter and wider than those in the other specimens.

### 3.3. Effect of Sol–Gel Impregnation Cycle on the Properties of Porous Bio-SiC Ceramics

**Figure 6:**
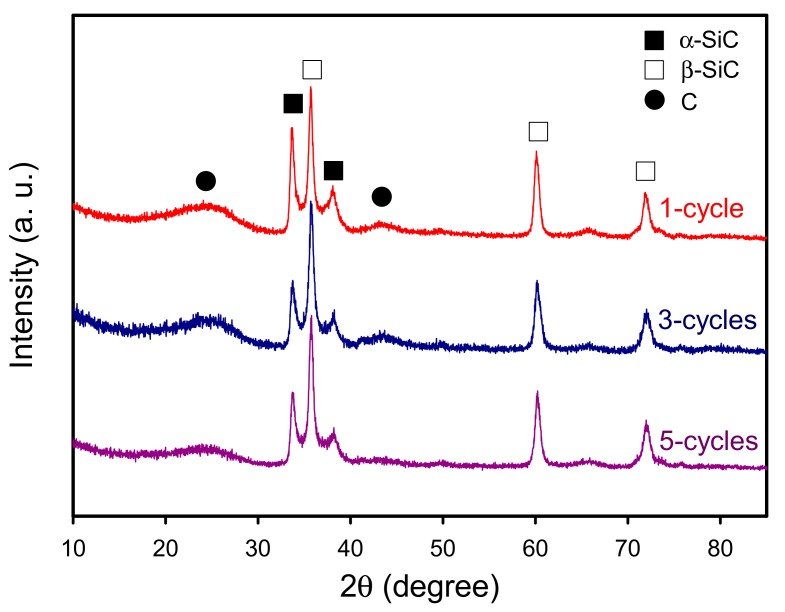
XRD patterns of sintered BcSiCs prepared using different repeated cycles of sol–gel impregnation process.

To further investigate the effect of sol–gel impregnation cycle on the properties of the derived porous bio-SiC ceramics, bamboo charcoal specimens impregnated with the SiO_2_ precursor sol in one, three, and five repeated cycles were analyzed. Figure 6 illustrates the XRD patterns of the bio-SiC ceramics that were prepared from the BcSiCs prepared using different sol–gel impregnation cycles. The XRD patterns did not show obvious differences among all bio-SiC ceramics. Peaks associated with β-SiC were observed at 2θ values of 35.3°, 60.0°, and 71.8°, along with three peaks associated with amorphous carbon. However, the intensity of the peaks associated with carbon decreased as the number of sol–gel impregnation cycles increased, and this finding is consistent with that reported by Qian et al. [18]. This phenomenon can be attributed to the fact that the amount of SiO_2_ in the BcSiCs increased with the number of impregnation cycles, which resulted in a more efficient conversion of charcoal to SiC ceramics during the carbothermal reduction reaction.

**Figure 7:**
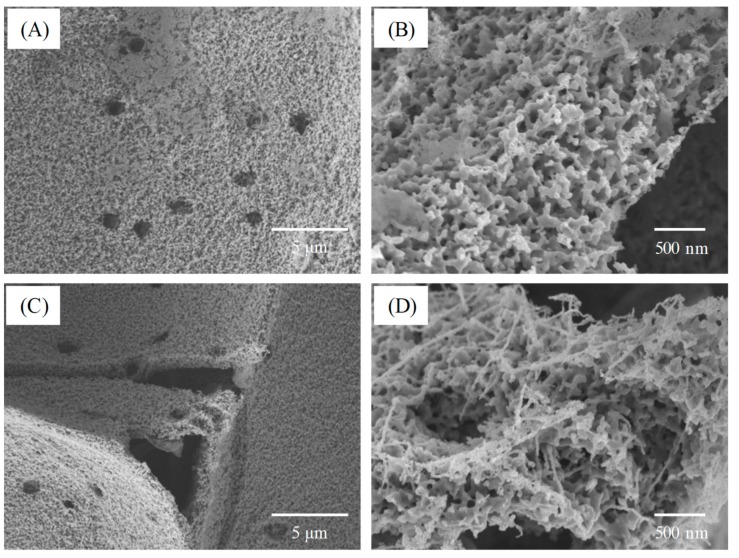
SEM micrographs of sintered BcSiCs prepared using three (**A**,**B**) and five repeated cycles (**C**,**D**) of sol–gel impregnation.

**Figure 8:**
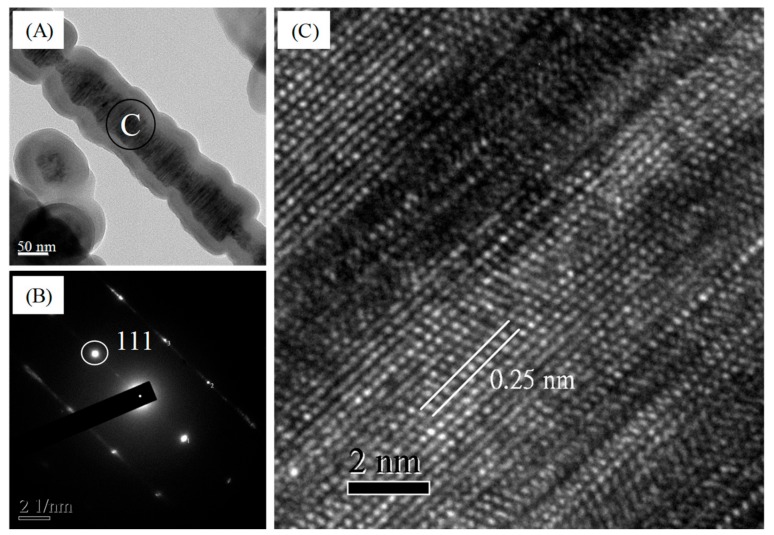
Transmission electron microscopy (TEM) image of a single β-SiC nanowire of sintered BcSiC prepared using five repeated cycles of sol–gel impregnation process (**A**), selected area electron diffraction (SAED) pattern (**B**), and high-resolution transmission electron microscopy (HRTEM) image (**C**) obtained for the β-SiC nanowire.

Figure 7 presents the microstructure of the bio-SiC ceramics prepared using three and five repeated cycles of sol–gel impregnation. The surface morphology of the cell walls of the bio-SiC ceramic prepared using three impregnation cycles (Figure 7A) was similar to that of the rough surface of the bio-SiC ceramic prepared using a single impregnation cycle (Figure 5C), except that SiC nanowires were not observed within the cell lumen. A foam-like cell wall was observed for the bio-SiC ceramic prepared using three impregnation cycles (Figure 7B). As reported by Qian and Jin [21], a possible reason for this observation is that CO was produced in addition to SiC during the reaction of carbon with SiO_2_. Moreover, the microstructure of the bio-SiC ceramic prepared using five impregnation cycles (Figure 7C,D) was similar to that of the bio-SiC ceramic prepared using three impregnation cycles, however, some SiC nanowires were formed on the surface of the cell wall of the bio-SiC ceramic. These results reveal that the microstructures of the bio-SiC ceramics did not differ significantly when the number of repeated sol–gel impregnation cycles exceeded three. Accordingly, the bio-SiC ceramics have higher porosity when the number of repeated sol–gel impregnation cycles exceeded three. Pastore et al. [38] reported that carbon foams with low density and high open porosity exhibit significant microwave absorption capabilities. Therefore, the bio-SiC ceramics may have high potential for electromagnetic applications when the number of repeated sol–gel impregnation cycles exceeded three. Figure 8 depicts the HRTEM images and selected area electron diffraction (SAED) patterns of SiC nanowires of the bio-SiC ceramics prepared using five impregnation cycles. The nanowires had a diameter of nearly 100 nm and a single crystal structure (Figure 8A). The (111) plane, representing the atomic plane perpendicular to the growth direction, of β-SiC (Figure 8B) had a planar distance of 0.25 nm (Figure 8C), indicating that the preferred growth direction of the nanowires was perpendicular to the (111) plane. A similar result was reported by Ding et al. [34].

**Figure 9:**
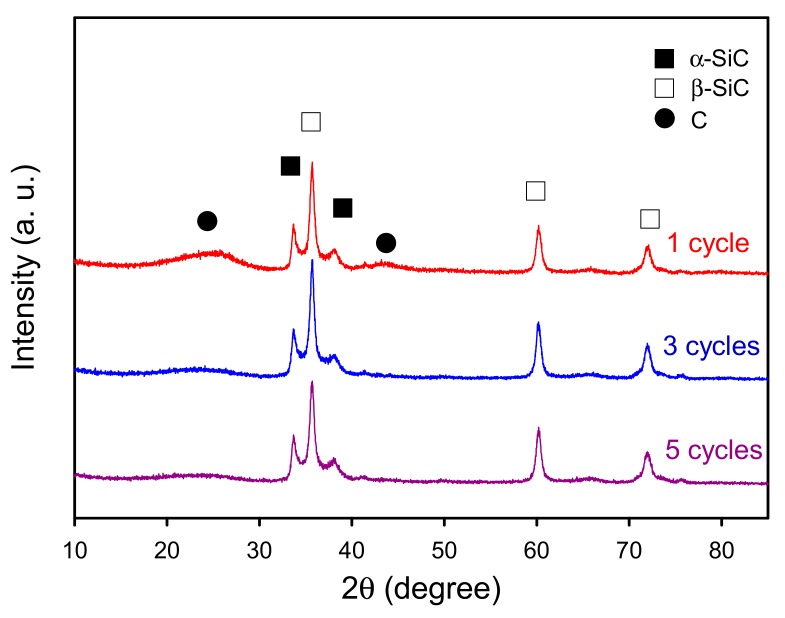
XRD patterns of sintered BSiCs prepared using different repeated cycles of sol–gel impregnation.

**Figure 10:**
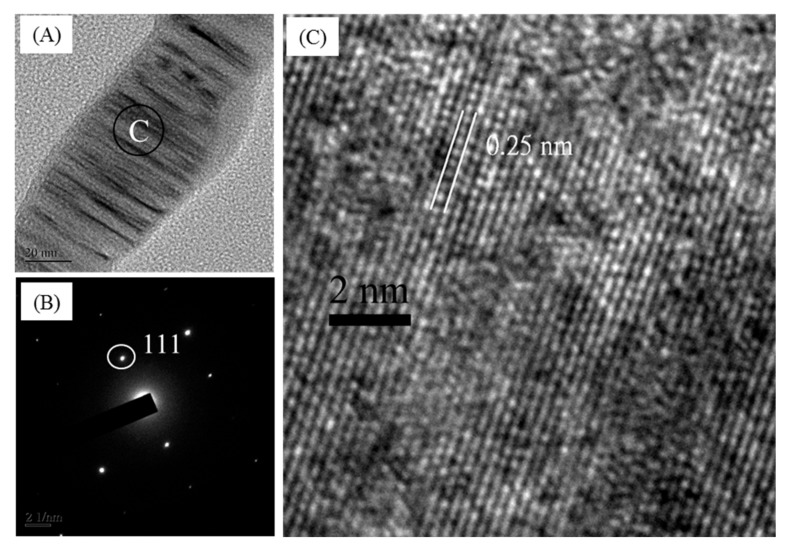
TEM image of a single β-SiC nanowire of sintered BSiC prepared using five repeated cycles of sol–gel impregnation (**A**), SAED pattern (**B**), and HRTEM image (**C**) observed for the β-SiC nanowire.

To further investigate the feasibility of directly producing bio-SiC ceramics from bamboo, BSiCs were prepared from air-dried bamboo by using MTMOS and a sol–gel process and then sintered at 1700 °C for 2 h in a nitrogen atmosphere. Figure 9 illustrates the XRD patterns of bio-SiC ceramics prepared from the BSiCs by using different sol–gel impregnation cycles. Similar to the XRD patterns of the sintered BcSiCs, β-SiC-associated peaks were observed at 2θ values of 35.3°, 60.0°, and 71.8°, in addition to peaks associated with carbon. The intensity of the peaks associated with carbon decreased as the number of repeated impregnation cycles increased. Figure 10 shows the HRTEM images and SAED patterns of a single SiC nanowire of a bio-SiC ceramic prepared from BSiCs using five impregnation cycles. The results in these images are similar to those obtained for the bio-SiC ceramic prepared from BcSiCs using five impregnation cycles. Accordingly, bio-SiC ceramics can be directly prepared from BSiCs, thus simplifying the manufacturing process of SiC ceramics. To the best of our knowledge, this is the first study to prepare biomorphic porous SiC ceramics directly from bamboo by combining sol–gel impregnation and carbothermal reduction.

## 4. Conclusions

In this study, bio-SiC ceramics were prepared by combining sol–gel impregnation and carbothermal reduction, with MTMOS and bamboo serving as the precursors. The results revealed that a higher sintering temperature resulted in a more complete reaction and thus, more thermally stable SiC. The resulting SiC ceramics comprised β-SiC and a trace amount of α-SiC. In addition, the microstructures of the cell walls of bio-SiC ceramics prepared using three and five impregnation cycles were determined to be foam-like (porous SiC) structures, in contrast to the microstructure of a bio-SiC ceramic prepared using a single impregnation cycle. Moreover, bio-SiC ceramics could also be successfully prepared from BSiC in this study, and their XRD patterns and HRTEM images were similar to those obtained for bio-SiC ceramics prepared from BcSiCs. According to a review of the literature, this is the first study to synthesize bio-SiC ceramics directly from bamboo using sol–gel and carbothermal reduction.
